# RNA m^6^A Modification Plays a Key Role in Maintaining Stem Cell Function in Normal and Malignant Hematopoiesis

**DOI:** 10.3389/fcell.2021.710964

**Published:** 2021-08-13

**Authors:** Peipei Wang, Mengdie Feng, Guoqiang Han, Rong Yin, Yashu Li, Shuxin Yao, Pengbo Lu, Yuhua Wang, Haojian Zhang

**Affiliations:** ^1^The State Key Laboratory Breeding Base of Basic Science of Stomatology and Key Laboratory of Oral Biomedicine Ministry of Education, School and Hospital of Stomatology, Wuhan University, Wuhan, China; ^2^Frontier Science Center for Immunology and Metabolism, School of Medicine, Medical Research Institute, Wuhan University, Wuhan, China; ^3^RNA Institute, Wuhan University, Wuhan, China

**Keywords:** RNA m^6^A, hematopoietic stem cells, leukemia stem cells, hematopoiesis, myeloid leukemia

## Abstract

N^6^-methyladenosine (m^6^A) is a commonly modification of mammalian mRNAs and plays key roles in various cellular processes. Emerging evidence reveals the importance of RNA m^6^A modification in maintaining stem cell function in normal hematopoiesis and leukemogenesis. In this review, we first briefly summarize the latest advances in RNA m^6^A biology, and further highlight the roles of m^6^A writers, readers and erasers in normal hematopoiesis and acute myeloid leukemia. Moreover, we also discuss the mechanisms of these m^6^A modifiers in preserving the function of hematopoietic stem cells (HSCs) and leukemia stem cells (LSCs), as well as potential strategies for targeting m^6^A modification related pathways. Overall, we provide a comprehensive summary and our insights into the field of RNA m^6^A in normal hematopoiesis and leukemia pathogenesis.

## Introduction

Hematopoietic homeostasis is maintained by rare multipotent hematopoietic stem cells (HSCs) via efficient self-renewal and differentiation into all lineage blood cells. This process is tightly controlled at multilayers such as the transcriptional and post-transcriptional levels (Orkin and Zon, 2008; Olson et al., 2020; Wilkinson et al., 2020). Alterations in these regulatory mechanisms affect the function of HSCs, and frequently cause hematologic diseases. Acute myeloid leukemia (AML) is an aggressive and fatal hematologic malignancy characterized by uncontrolled expansion of poorly differentiated myeloid cells (Dohner et al., 2015), and its development is associated with accumulation of acquired genetic and epigenetic changes in hematopoietic stem/progenitor cells (HSPCs) (Cancer Genome Atlas Research Network Ley et al., 2013; Shlush et al., 2014). These alterations confer HSPCs with increased self-renewal capacity and impair their normal differentiation trajectory, thereby subsequently transforming HSPCs into leukemia stem cells (LSCs) (Lu et al., 2016; Yang et al., 2016). The existence of LSCs is also considered as the main reason for AML relapse. Therefore, exploring the underlying mechanisms of maintaining the function of HSCs and LSCs is always an attractive topic in this field.

The post-transcriptional modification of RNA plays important roles in regulating gene expression. m^6^A was first discovered in the 1970s, and is the most abundant one among about 160 chemical marks on cellular RNA that have been discovered to date (Desrosiers et al., 1974; Helm and Motorin, 2017). About one-third of mammalian mRNAs have been identified as containing m^6^A modification with an average of 3–5 modifications per mRNA (Huang et al., 2020). Along with the development of m^6^A high-throughput sequencing technology that enables profiling of m^6^A modifications at the transcriptome-wide level, it is found that m^6^A modification sites have a typical consensus motif DRACH and are mainly enriched in the coding sequence and 3’untranslated region (Dominissini et al., 2012; Meyer et al., 2012). Moreover, m^6^A modification is reversible and dynamically controlled by m^6^A modifiers including writers, erasers and readers, and plays key roles in determining RNA fate by regulating RNA processing such as decay, stability, splicing, transportation, and translation. Increasing evidences including studies from our laboratory indicate that RNA m^6^A is involved in many biological processes, including hematopoiesis and leukemogenesis (Vu et al., 2019; Wang et al., 2020). In this review, we summarize the roles of m^6^A writers, readers and erasers in normal hematopoiesis and acute myeloid leukemia by focusing on their function in HSCs and LSCs maintenance, as well as potential strategies for targeting m^6^A modification related pathways.

## Regulation of RNA m^6^A Modification

**FIGURE 1:**
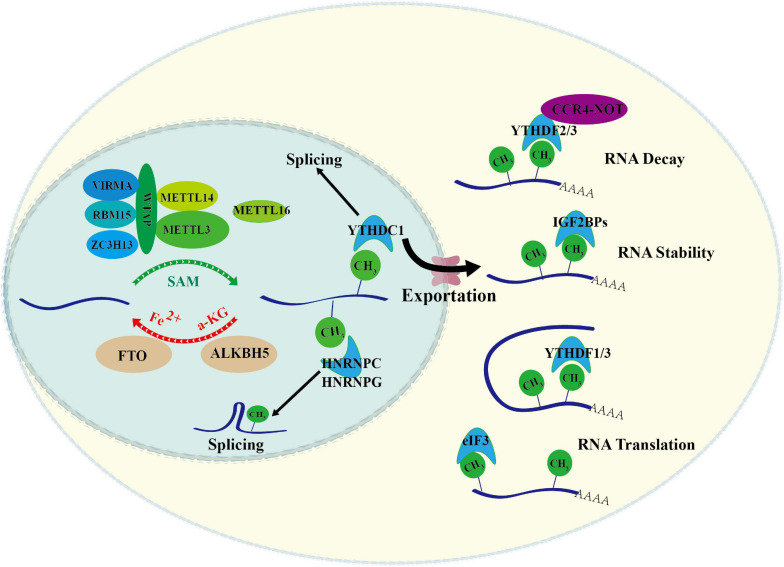
Overview of RNA m^6^A modification. m^6^A methylation is catalyzed by the writer complex that is composed by METTL3, METTL14, WTAP, METTL16, VIRMA, RBM15, ZC3H13, and KIAA1429. m^6^A methylation is removed by erasers including ALKBH5 and FTO. The m^6^A-modified RNA reader proteins include YTHDF1/2/3, YTHDC1/2, IGF2BP1/2/3, and HNRNPs. These readers bind to the target RNA in an m^6^A-dependent manner, and affects the metabolic processes of RNA, such as pre-mRNA splicing, nuclear export, RNA decay, RNA stability and RNA translation.

RNA m^6^A is a dynamic and reversible modification that is executed by m^6^A modifiers and related factors, which can be divided into three different functional groups, writers, erasers, and readers. The writers are responsible for installing m^6^A marks to RNA, the erasers selectively remove m^6^A marks, and the readers recognize m^6^A marks specifically. m^6^A methyltransferase complex, also called “m^6^A writer,” is composed of methyltransferase-like 3 (METTL3), METTL14, WT-associated protein (WTAP), RBM15/RBM15B, KIAA1429, and ZC3H13 (Liu et al., 2014; Ping et al., 2014; Knuckles et al., 2018; Wen et al., 2018; Hu et al., 2020). m^6^A erasers, FTO and ALKBH5, are the main demethylases (Jia et al., 2011; Zheng et al., 2013). m^6^A modifications are recognized by different m^6^A readers, including YTH domain-containing protein 1–2 (YTHDC1/2), YTH domain-containing family member 1–3 (YTHDF1/2/3), and insulin-like growth factor-2 mRNA-binding protein (IGF2BP) family IGF2BP1/2/3 (Theler et al., 2014; Xiao et al., 2016; Hsu et al., 2017; Huang et al., 2018). They closely collaborate in controlling m^6^A modification and determining RNA fates (Figure 1).

### Writer of RNA m^6^A

Writer of m^6^A, the methyltransferase complex, is responsible for catalyzing the transfer of methyl group from S-adenosylmethionine (SAM) to the sixth N atom of RNA adenosine. It is currently believed that METTL3 is the only catalytical protein in this complex, whereas METTL14 maintains the activity and structural stability of METTL3/METTL14 heterodimer (Wang P. et al., 2016). METTL3 contains the leading helix structure (LH) domain, nuclear localization signal (NLS) domain, CCH-type zinc finger domain (ZFD), and two SAM structure binding domains–methyltransferase domain (MTD). LH and NLS mediate the interaction of METTL3 with METTL14 (Liu et al., 2014; Wang P. et al., 2016; Wang X. et al., 2016). ZFD serves as the target recognition domain and fulfills the methyltransferase activity of the METTL3-METTL14 complex (Huang J. et al., 2019).

The activity and specificity of m^6^A writer also relies on the subunits of this complex. As an important subunit of m^6^A writer complex, WTAP promotes METTL3-METTL14 heterodimer to enter the nuclear plaque and stabilize it (Ping et al., 2014). Biochemical experiments have proved that WTAP protein alone does not function as a methyltransferase *in vitro* but as an indispensable subunit for the complex. RBM15 is another important subunit and facilitates the recruitment of m^6^A writer complex to specific RNA (Patil et al., 2016). ZC3H13 exists in an evolutionarily conserved complex ZC3H13-WTAP-Virilizer-Hakai. ZC3H13 loss promotes the transfer of WTAP from nucleus to cytoplasm accompanying with decreased nuclear METTL13 and METTL14, thus blocking m^6^A installation (Knuckles et al., 2018; Wen et al., 2018). VIRMA contains the RBP domain, and facilitates m^6^A writer complex to the 3′UTR region, suggesting that VIRMA plays a significant role in m^6^A modification concentrated near 3′UTR and stop codon (Yue et al., 2018). Together, RNA m^6^A installation is a complicated process, and multiple factors involved in this process guarantee its accuracy.

### Erasers of RNA m^6^A

Currently, two m^6^A demethylases have been reported, FTO and ALKBH5, and both belong to the ALKB family (Jia et al., 2011; Zheng et al., 2013). Although FTO is a member of the ALKB family, its C-terminus has a specific domain (long loop) that can demethylate methylated bases (Zhang et al., 2019). Beyond m^6^A, FTO can also catalyze the demethylation of m^6^Am on mRNA and m^1^A on tRNA. Recent study showed that the cellular distribution of FTO is distinct among different cell lines, which affects the access of FTO to different RNA substrates. FTO in the nucleus has a higher affinity for m^6^A, while FTO in the cytoplasm has a higher affinity for m^6^Am (Wei et al., 2018). Although a nuclear localization signal at the N-terminus guides FTO into the nucleus, factors that influence FTO location remain unknown yet. ALKBH5 is another demethylated enzyme that specifically recognizes the m^6^A on RNA. Knockout of ALKBH5 in mice does not affect the health status except for the defect of spermatogenesis in mice, which makes ALKBH5 more suitable as a therapeutic target (Zheng et al., 2013; Shen et al., 2020; Wang et al., 2020). Notably, α-KG and Fe^2+^ are essential for the demethylation activity of FTO and ALKBH5, however, it remain elusive how the activities of these m^6^A erasers are regulated in different contexts.

### Readers of RNA m^6^A

Identification of m^6^A readers has provided important information about how m^6^A acts in determining RNA fates, and the list of m^6^A readers is still expanding. Both YTHDF1/2/3 and YTHDC1/2 can be grouped into one class, as they all contain the same YTH domain that is responsible for recognizing m^6^A. YTHDF2 mainly promotes degradation of m^6^A-tagged mRNAs (Du et al., 2016), while YTHDF1 and YTHDF3 affect the translation of their target transcripts (Shi et al., 2017). The different consequences brought by these different readers may result from their associated regulatory machinery or related factors. For instance, YTHDF1 interacts with eukaryotic initiation factor 3 (eIF3) in the 48S translation initiation complex and further recruits other translational factors for facilitating translation (Lee et al., 2015); YTHDF3 recruits eIF4G2 directly bound to internal ribosome entry sites to initiate eIF4E-independent translation (Yang et al., 2017; Di Timoteo et al., 2020); YTHDF2 recruits CCR4-NOT deadenylase complex for mRNA decay (Du et al., 2016). Similarly, YTHDC1/2 executes a distinct function in fine-tuning m^6^A-tagged transcripts, although they share the same sequence pattern with YTHDF1/2/3. YTHDC1 mainly locates in the nucleus and participates in m^6^A-tagged mRNA export from the nucleus to the cytoplasm (Zhang et al., 2010; Xu et al., 2014; Xiao et al., 2016; Roundtree et al., 2017). Recent studies indicate that YTHDC1 also mediates mRNA splicing by recruiting two competitive mRNA splicing factors serine/arginine-rich splicing factor 3 (SRSF3) and SRSF10 (Xiao et al., 2016). YTHDC2 affects the stability and translation of mRNA by recognizing m^6^A modification (Xu et al., 2015; Wojtas et al., 2017; Kretschmer et al., 2018).

IGF2BPs form another group of m^6^A readers as they use common RNA binding domains (RBDs) for recognizing m^6^A-tagged RNA. IGF2BPs contains 4 repetitive KH protein domains, and the third and fourth KH protein domains are essential for IGF2BPs to recognize m^6^A. IGF2BPs mainly regulate the stability of their target mRNA in an m^6^A-dependent manner, and in Hela cells they share about 60% target transcripts (Huang et al., 2018). But the regulatory machinery of IGF2BPs remains elusive. Our recent work revealed that YBX1 cooperates with IGF2BPs to promote the stability of m^6^A-tagged transcripts (Feng et al., 2021), suggesting YBX1 is one component of IGF2BP regulatory machinery. However, what the other factors are involved in the machinery still needs investigation in the future. Several heterogeneous nuclear ribonucleoproteins (HNRNPs) including HNRNPC, HNRNPG, and HNRNPA2/B1 also function as m^6^A reader. For instance, HNRNPA2B1 can directly bind to the primary miRNA (pri-miRNA) that carry m^6^A mark, and promotes the processing of the mature of pri-miRNA through cooperating with miRNA microprocessing complex DGCR8 (Alarcón et al., 2015).

### Open Scientific Questions

The biology of RNA modification has attracted burst interests, and the important role of m^6^A modification in regulating RNA fates has been appreciated. While the field become flourishing, some key scientific questions remain unknown. First, how are the activities of m^6^A modifiers regulated? Changing the expression levels of m^6^A modifiers in different cells may be a major approach for fine-tuning their activities. For instance, increased expression of several m^6^A modifiers such as METTL3, FTO, and ALKBH5 have been observed in leukemia cells (discussed in the following) (Li et al., 2017; Vu et al., 2017; Wang et al., 2020). In addition, the activities of m^6^A modifiers may be affected by metabolites. Recent study showed that oncometabolite 2-hydroxyglutarate (2HG) inhibits FTO (Su et al., 2018). Moreover, the cellular distribution and post-translational modification of m^6^A modifiers also regulate their activities (Lin et al., 2016; Du et al., 2018; Wei et al., 2018; Sun et al., 2020). Second, what determines the transcript specificity of m^6^A modifiers? It is known that m^6^A is installed on mRNA co-transcriptionally by m^6^A writer complex (Yang et al., 2018), but it is still unknown how the transcripts are selected by m^6^A writer. Same question is also applicable to m^6^A erasers and readers. Increasing evidence shows that diverse regulatory machinery can be recruited to m^6^A-tagged mRNA through m^6^A readers, and RBPs involved in these machineries might convey specificity of m^6^A readers toward certain m^6^A sites or m^6^A-tagged RNA. Thus, identifying the cofactors of m^6^A modifiers may be more important in the future. Last, we should appreciate the importance of context issue in RNA modification biology. From this point, it becomes reasonable that RNA m^6^A is highly dynamic and context-dependent.

## RNA m^6^A in Maintaining the Function of HSCs and LSCs

**FIGURE 2:**
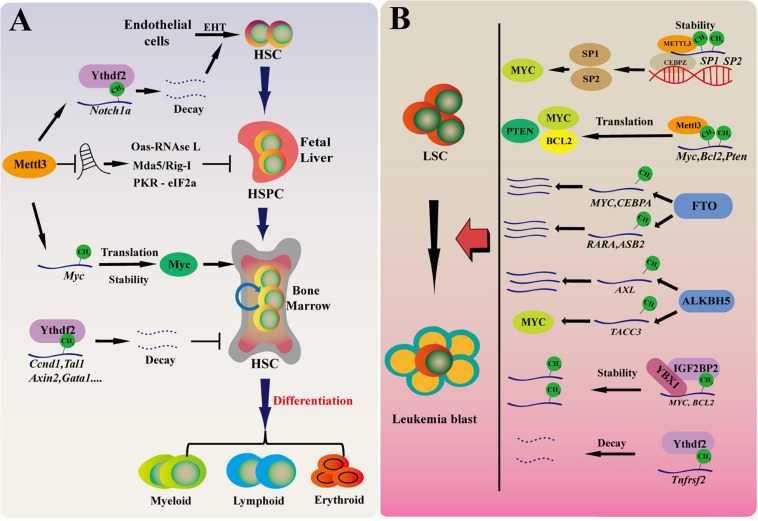
RNA m^6^A in maintaining HSCs and LSCs function in normal and malignant hematopoiesis. **(A)** m^6^A modification regulates normal hematopoietic system. Mettl3 mediated m^6^A modification regulates HSPC fate specification by inhibiting Notch signaling during early definitive hematopoiesis. During HSPC expansion in fetal liver, Mettl3 deletion promotes dsRNA formation, activates OAS RNase L and PKR-eIF2a pathways, and upregulates MDA5/RIG-I, leading to hematopoietic failure. In adult HSCs, Mettl3 regulates the translation of c-Myc and promotes the proliferation of hematopoietic stem cells. Ythdf2-deficient HSCs display chronic activation of inflammatory pathways, resulting in a progressive myeloid bias. EHT, endothelial-to-hematopoietic transition. **(B)** The role of RNA m^6^A for LSCs in myeloid leukemia. The m^6^A level of cancer-related genes are affected with m^6^A “writer,” “reader,” and “eraser” by regulating mRNA translation, decay, stability in the development of AML. These cancer-related genes promote the development of leukemia by affecting proliferation, differentiation, cancer self-renewal, and apoptosis.

HSCs locate at the top of the hematopoietic hierarchy and are responsible for replenishing blood system throughout life. LSCs transformed from normal HSCs initiate the development of myeloid leukemia such as AML. Increasing evidence show that RNA m^6^A plays key roles in sustaining the function of HSCs and LSCs in normal and malignant hematopoiesis. In the following, we summarize the research advances in this field (Figure 2).

### RNA m^6^A Is Essential for HSC Maintenance in Normal Hematopoiesis

Both the emergence and functional maintenance of HSCs in hematopoiesis are delicately controlled. HSCs are derived early from embryonic precursors, such as hemogenic endothelial cells and pre-hematopoietic stem cells (Zhou et al., 2016). The first HSC appears in mid-gestational mouse embryos at embryonic day 10.5 (E10.5), and progressively colonizes fetal liver and dramatically expands there from E12. Before birth, HSCs migrate into bone marrow, where they maintain the homeostasis of hematopoietic system by extensively self-renewing and differentiating into entire blood lineages throughout lifetime.

RNA m^6^A modification controls the fate determination of HSCs and progenitor cells during vertebrate definitive hematopoiesis. Using a zebrafish model and deleting Mettl3 expression by morpholino (MO), a recent study found that HSPCs differentiation toward erythroid, myeloid, and lymphoid cells is substantially impaired in *mettl3* morphants, while the primitive hematopoiesis is relatively unaltered (Zhang et al., 2017). Mettl3 loss abolishes the transition of endothelial cells into HSCs, as showing the decreased number of hemogenic endothelium and emerging HSPCs in *mettl3* morphants. They investigated the molecular mechanisms by integrating RNA-seq with m^6^A-seq, and found that deletion of mettl3 in endothelial cells upregulates the Notch and vascular endothelial growth factor pathways, and identified that notch1a as one of the downstream targets of mettl3. They confirmed that Mettl3-mediated m^6^A modification regulates HSPC generation through inhibiting endothelial Notch signaling, which is mediated by m^6^A reader Ythdf2 (Zhang et al., 2017). Thus, m^6^A modulates HSPC specification in early definitive hematopoiesis of zebrafish embryogenesis. Recent studies showed that Mettl3 is also essential for mammalian hematopoiesis (Gao et al., 2020; Jiang et al., 2021). Deletion of *Mettl3* and m^6^A in *Vav-Cre;Mettl3^*fl/fl*^* mice caused hematopoietic failure with expansion of phenotypical Lin^–^Sca-1^+^c-Kit^+^ (LSK) HSPCs in the fetal liver that were functionally defective. Further analysis revealed that loss of m^6^A results in robust transcriptional upregulation of interferon-stimulated genes (ISGs) and 2′, 5′-oligoadenylate synthetase (*Oas*) genes and induces a dsRNA-mediated innate immune response, showing activation of the OAS-RNase L and PKR-eIF2a pathways and upregulation of the dsRNA sensors MDA5 and RIG-I (Gao et al., 2020). These studies reveal that RNA m^6^A plays key role in embryonic hematopoiesis.

In adult hematopoiesis, m^6^A is essential for maintaining HSC function. Inducible deletion of *Mettl3* in adult hematopoietic system in *Mx1-Cre;Mettl3^*fl/fl*^* mice does not significantly change the production ratio of mature myeloid cells, but blocks the normal differentiation and causes accumulation of phenotypical HSCs with long-term hematopoietic disorder and impaired hematopoietic reconstitution potential (Yao et al., 2018; Cheng et al., 2019; Lee et al., 2019). Knockdown of METTL3 or METTL14 by shRNAs can also significantly inhibit the proliferation and promote the differentiation of human umbilical cord blood derived CD34^+^ HSPCs (Vu et al., 2017; Martin and Park, 2018). Using single-cell RNA-seq in combination with transcriptomic profiling of HSPCs, researchers found that m^6^A-deficient HSCs fails to symmetrically differentiate due to alteration of *Myc* mRNA abundance, and enforced expression of Myc rescues differentiation defect of *Mettl3*-deficient HSCs (Cheng et al., 2019; Lee et al., 2019). Thus, these studies reveal a key role of m^6^A in governing HSC differentiation in adult hematopoietic system, which is distinct to its function in embryonic hematopoiesis, indicating a developmental stage-specific requirement for m^6^A in hematopoiesis.

Interestingly, recent works further add the complexity about the role of m^6^A in hematopoiesis. Two early studies found that YTHDF2 depletion significantly expands hematopoietic stem cells in mouse and human umbilical cord blood without skewing lineage differentiation preference or leading to hematopoietic malignancy (Li et al., 2018; Paris et al., 2019). Intriguingly, Mapperley et al. (2021) recently analyzed the long-term effect of Ythdf2 deletion on HSC maintenance and multilineage hematopoiesis. They found that HSCs from young mice with *Ythdf2* deficiency cannot be transplanted continuously. Furthermore, *Ythdf2*-deficient HSCs displays chronic activation of inflammatory pathways, resulting in a progressive myeloid bias, loss of lymphoid potential and HSC expansion with functional defect of long-term reconstitution (Mapperley et al., 2021). Of course, the roles of other m^6^A readers in hematopoiesis remain unknown. However, it becomes clear that m^6^A eraser AlKBH5 is not dispensable for adult hematopoiesis and HSC function. Using Mx1-Cre to conditional delete *Albkh5* in mouse hematopoietic cells, we found that Alkbh5 deficiency does not affect normal hematopoiesis. We also performed serial transplantation and confirmed that loss of Alkbh5 does not affect HSCs self-renewal, differentiation and long-term hematopoietic function (Shen et al., 2020; Wang et al., 2020). Knockdown of ALKBH5 also does not affect the colony forming ability of HSPCs derived from human umbilical cord blood (Wang et al., 2020). We speculate that FTO, another m^6^A eraser, may compensate the function of Alkbh5 in *Alkbh5*-deficient cells, thus loss of Alkbh5 alone does not significant affect normal hematopoiesis and HSC function. Overall, we believe that m^6^A acts much more complex role in hematopoiesis (Figure 2A).

### The Role of RNA m^6^A for LSCs in Myeloid Leukemia

Most myeloid leukemia is initiated by LSCs that are transformed from dysregulated HSCs. Similarly, RNA m^6^A also plays essential roles in leukemia and LSC function. Compared with normal HSPCs or other types of tumor cells, the expression level of METTL3 is obviously higher in AML (Barbieri et al., 2017; Vu et al., 2017). Overexpression of METTL3 inhibits leukemia cell differentiation and increases cell growth; conversely, deletion of METTL3 in human myeloid leukemia cells can induce cell differentiation and promote apoptosis, and delay *in vivo* leukemia development (Barbieri et al., 2017; Vu et al., 2017; Guirguis et al., 2020). Single-nucleotide-resolution mapping coupled with RNA-seq and ribosome profiling revealed that m^6^A promotes the translation of downstream targets including *c-MYC*, *BCL2*, and *PTEN* (Vu et al., 2017). METTL3 may act in another way. In another study, ChIP-seq experiment showed that METTL3, independently of METTL14, binds to chromatin and localizes to transcriptional start site (TSS) of active genes. Promoter-bound METTL3 recruits CEBPZ and regulates the translation of downstream oncogenic drivers SP1 and SP2. SP1 in turn regulates the expression of c-MYC and ultimately promotes the occurrence and development of leukemia (Barbieri et al., 2017). Another subunit of m^6^A writer core complex, METTL14 is also highly expressed in AML cells and is required for leukemia. In mechanism, transcription factor SPI1 negatively regulates METTL14 expression, and MYB and MYC are functional downstream targets of METTL14 (Weng et al., 2018).

Actually, FTO is the first m^6^A modifier that is reported to play an oncogenic role in AML (Li et al., 2017). FTO is highly expressed in leukemia cells from different subtypes of AML, and especially in leukemia stem cells. FTO knockout or inhibition can significantly inhibit the self-renewal of leukemia stem cells, thus hindering the occurrence and development of AML; conversely, high expression of FTO promotes the growth of leukemia cells and accelerates leukemogenesis. Mechanistically, FTO regulates the degradation of *ASB2* and *RARA* mRNA in an m^6^A-dependent manner (Li et al., 2017). IDH1/2 catalyze the oxidative decarboxylation of isocitrate to α-ketoglutarate (α-KG). IDH1/2 mutations occur in about 20% of AML, resulting in increased production of R-2-hydroglutarate (R-2HG). Thus, R-2HG is considered as an oncometabolite. Interestingly, as R-2HG is structurally close to α-KG, it competitively inhibits the activity of α-KG-dependent dioxygease, FTO, thereby increasing the overall level of m^6^A without affecting FTO expression and decreasing the stability of *MYC* and *CEBPA* mRNA in R-2HG-sensitive leukemia cells (Su et al., 2018). Moreover, a recent study found that R-2HG also suppresses glycolysis in leukemia cells by abrogating FTO/m^6^A/YTHDF2-mediated upregulation of two critical glycolytic genes *phosphofructokinase platelet (PFKP)* and *lactate dehydrogenase B (LDHB)* (Qing et al., 2021). Thus, these works provide rationale for FTO as a potential therapeutic target for AML treatment.

ALKBH5 is regarded as the major demethylase for most mRNA m^6^A, as FTO only demethylates 5–10% of mRNA m^6^A in common cells (maybe up to 40% in some AML cells). Recent works from our group and Dr. Chen’s group simultaneously revealed the key and selective role of ALKBH5 in self-renewal and maintenance of leukemia stem cells (Shen et al., 2020; Wang et al., 2020). We found that ALKBH5 expression is regulated by chromatin state alteration during leukemogenesis of human AML, which is mediated by histone demethylase KDM4C. KDM4C reduces H3K9me3 levels and promotes recruitment of MYB and Pol II to *ALKBH5* promoter region (Wang et al., 2020). Using MLL-AF9 mouse model and a series of leukemia reconstitution experiments in the *Alkbh5* conditional knockout mice, we confirmed that *Alkbh5* deletion could significantly inhibit the occurrence and development of AML and prolong the survival time of the knockout mice. Furthermore, integration of m^6^A-seq, SLAM-seq, Ribo-seq data analysis showed that ALKBH5 knockdown significantly increases the overall level of m^6^A modification, decreases the stability of the overall mRNA, and does not affect the overall mRNA translation. We focused on a receptor tyrosine kinase AXL and found that ALKBH5 affects *AXL* mRNA stability in an m^6^A-dependent way. AXL belongs to the TAM (TYRO3, AXL, MER) receptor kinase family, and has been reported that AXL can phosphorylate FLT3 in AML to promote the pathological progress of AML. At the same time, AML patients with high expression of AXL have poor prognosis (Park et al., 2015). We confirmed that AXL activates downstream PI3K/AKT pathway in AML and mediates the function of ALKBH5 in AML. Meanwhile, Cheng et al. (2020) and Shen et al. (2020) found that ALKBH5 knockdown accelerates the degradation of *TACC3* mRNA, an oncogenic factor described to be critical in the growth of various cancer cells. These two complimentary studies clearly uncover the important role of ALKBH5 in the pathogenesis of AML and LSCs maintenance, but has no significant effect on normal hematopoietic differentiation. The findings from our group also link chromatin state dynamics with expression regulation of m^6^A modifiers.

The roles of other m^6^A modifiers in LSC maintenance and leukemogenesis are also being recognized recently. It has been reported that WTAP is highly expressed in AML and is associated with poor prognosis. Knockdown of WTAP inhibits cell proliferation, induces apoptosis and enhances myeloid differentiation, and plays a oncogenic role in leukemia (Bansal et al., 2014; Naren et al., 2021). Importantly, upregulation of WTAP is not enough to promote the proliferation of leukemia cells when the function of METTL3 is absent, indicating that the oncogenic role of WTAP depends on METTL3 and RNA m^6^A (Sorci et al., 2018). Paris et al. (2019) found that YTHDF2 is highly expressed in AML and is required for disease initiation as well as propagation in mouse and human AML. Ythdf2 loss decreases the half-life of diverse m^6^A transcripts that contribute to the overall integrity of LSC function, including the tumor necrosis factor receptor Tnfrsf2, whose upregulation in *Ythdf2*-deficient LSCs primes cells for apoptosis (Paris et al., 2019). IGF2BPs promote mRNA stability and translation (Huang et al., 2018). It has been found that IGF2BP1 affects the proliferation and tumorigenic potential of leukemia cells by regulating the key factors of self-renewal, such as HOXB4, MYB, and ALDH1A1 (Elcheva et al., 2020; Schuschel et al., 2020). Overall, while the function of the remaining m^6^A modifiers in hematologic malignancies is being exploited, the studies so far clearly show that RNA m^6^A is essential for the development of myeloid leukemia and LSCs maintenance (Figure 2B).

### Targeting m^6^A Modifiers: A Promising Therapeutic Strategy for Myeloid Leukemia

Current studies have established a rationale for developing therapeutic approaches against leukemia by targeting RNA m^6^A modifiers. In a recent study, via a high throughput screen of 250,000 diverse drug-like compounds, a highly potent and selective first-in-class catalytic inhibitor of METTL3, STM2457, was identified (Yankova et al., 2021). STM2457 can directly bind to the SAM binding site of METTL3, thereby blocking its methyltransferase activity and affecting the translation of BRD4, c-Myc, SP1 and other genes. STM2457 treatment significantly inhibits proliferation, induces differentiation and increase apoptosis of leukemia cells. Surprisingly, STM2457 has no significant effect on normal HSCs and other normal cells, although previous studies showed that genetic deletion of METTL3 impairs HSC differentiation and damage normal hematopoiesis (Vu et al., 2017; Martin and Park, 2018). This study may present a promising targeting strategy for AML treatment. Notably, given that METTL3 is the core subunit of RNA m^6^A writer complex and is essential for various physiological processes, it needs to pay much attention to the possible side effects resulting from targeting METTL3 in the future.

FTO is another potential target for cancer therapy, and small molecules against FTO are being developed. Meclofenamic acid (MA) is an inhibitor of FTO (Huang et al., 2015), and based on the structural principles underlying FTO/MA interaction, recently. Huang Y. et al. (2019) developed new FTO inhibitors FB23 and FB23-2, and found that FB23-2 displays inhibitory effects on leukemia cells *in vitro* and *in vivo*. They further conducted a structure-based virtual screening of the 260,000 compounds, and identified two small molecule inhibitors CS1 (bisantrene) and CS2 (brequinara). Inhibition of FTO by these two compounds attenuates LSC self-renewal and reprograms immune response (Su et al., 2020). The inhibition of immune escape of leukemia cells with targeting FTO is mainly associated with the immunosuppressive molecule LILRB4, which is highly expressed in leukemia cells. These studies may provide a model for identifying potential inhibitors for m^6^A modifiers. In the future, it is necessary to further clarify the role of FTO in normal hematopoiesis and HSCs. Strikingly, recent studies of our group and Dr. Chen’s group have clearly showed that ALKBH5 is essential for AML stem cells but dispensable for normal HSCs and hematopoiesis, which signify ALKBH5 as a novel target for AML treatment. In addition, genetic deletion of Alkbh5 has no significant effect on the whole life span and physiological status of mice except spermatogenesis (Zheng et al., 2013). From this point, we believe ALKBH5 might be another very promising therapeutic target.

### Open Scientific Questions

It becomes clear that RNA m^6^A modification and the related modifiers play critical roles in maintaining stem cell function in normal and malignant hematopoiesis. However, some key scientific questions remain unclear. For instance, in term of definitive hematopoiesis during mammalian embryogenesis, which developmental stages are really affected by RNA m^6^A? Why does m^6^A play distinct roles in embryonic and adult hematopoiesis? What is the underlying mechanism? Interestingly, some m^6^A modifiers are selectively essential for LSCs and leukemia but are dispensable for normal hematopoiesis. What is the underlying rationale for these different effects on LSCs and normal HSCs of certain m^6^A modifiers, such as ALKBH5? Moreover, genomic instability is one of main mechanisms for cancer development and progression, and various genetic deletions, insertions or chromosome translocations have been found in leukemia. Recent studies have shown that m^6^A involves in DNA damage repair and genomic stability (Xiang et al., 2017; Zhang et al., 2020), it will be interesting to explore whether RNA m^6^A modification regulates genomic instability during leukemogenesis. Rapidly accumulating evidence also shows the crosstalk between RNA methylation and histone/DNA epigenetic mechanisms (Huang H. et al., 2019; Liu et al., 2020; Wei and He, 2021), which guides the recruitment of chromatin modifiers or RNA m^6^A machinery, and regulates the transcriptional activity and translation. Thus, further exploring the underlying mechanisms might provide more information for the specification of RNA m^6^A in normal and malignant hematopoiesis. In addition, m^6^A writers and erasers are all required for hematologic malignancies, and deletion of either one results in similar phenotypes. From the biochemical or molecular view, m^6^A writers and erasers act in totally opposite ways in m^6^A modification, why do they function similarly at the pathological context? We propose that m^6^A plays a protective role under stress setting, such as oncogene transformation, which induces higher level of transcription during leukemogenesis. In response to this higher oncogenesis-induced transcriptional stress, cells strengthen the regulatory networks including RNA m^6^A pathway to maintain the homeostasis of RNA metabolism. Thus, interfering RNA m^6^A pathway by altering either m^6^A writers or erasers results in the similar cellular consequence under pathogenesis. Overall, these questions need to be addressed in the future.

## Conclusion and Perspectives

In summary, accumulated evidences in the past few years show that m^6^A modification plays essential role in normal hematopoiesis and leukemia pathogenesis. However, the underlying molecular mechanisms are still unclear. How does RNA m^6^A specifically and precisely regulate the physiological and pathological processes of hematopoiesis. RBPs such as YBX1 regulate the interaction between m^6^A-tagged RNA substrates and m^6^A modifiers (Feng et al., 2021), suggesting that RBPs or cofactors for m^6^A modifiers may act key roles in determining the precision and specificity of RNA m^6^A. In addition, it is necessary to investigate the dynamics of RNA m^6^A profiling during normal and malignant hematopoiesis. Unfortunately, current approaches of m^6^A-seq are not suitable for rare stem cells. Therefore, developing a highly sensitive m^6^A-seq is urgent and very useful in the future. Given the important role of RNA m^6^A in AML, it will be very exciting to explore the therapeutic potentials and clinical benefit by targeting m^6^A modifiers in AML treatment. Indeed, small molecule compounds that target m^6^A regulators (METTL3 and FTO) have been developed (Qiao et al., 2016; Huang Y. et al., 2019; Yankova et al., 2021). In the next, developing effective therapeutic strategies by targeting RNA m^6^A pathway and clarifying their feasibility in the clinical should be paid much attention. Taken together, these studies shed light on the role of RNA m^6^A in normal and malignant hematopoiesis, and we believe this just opens the door for us to explore the unknown RNA modification world.

## Author Contributions

PW, MF, GH, and HZ wrote the manuscript. GH prepared the figures. RY, YL, SY, PL, and YW contributed to critically discussing the manuscript. All authors contributed to the article and approved the submitted version.

## Conflict of Interest

The authors declare that the research was conducted in the absence of any commercial or financial relationships that could be construed as a potential conflict of interest.

## Publisher’s Note

All claims expressed in this article are solely those of the authors and do not necessarily represent those of their affiliated organizations, or those of the publisher, the editors and the reviewers. Any product that may be evaluated in this article, or claim that may be made by its manufacturer, is not guaranteed or endorsed by the publisher.
